# Limits to modern contraceptive use among young women in developing countries: a systematic review of qualitative research

**DOI:** 10.1186/1742-4755-6-3

**Published:** 2009-02-19

**Authors:** Lisa M Williamson, Alison Parkes, Daniel Wight, Mark Petticrew, Graham J Hart

**Affiliations:** 1 MRC Social and Public Health Sciences Unit, 4 Lilybank Gardens, Glasgow, G12 8RZ, UK; 2 Public and Environmental Health Research Unit, Keppel Street, London School of Hygiene and Tropical Medicine, London, WC1E 7HT, UK; 3 Centre for Sexual Health and HIV Research, Research Department of Infection and Population Health, University College London, Mortimer Market Centre, off Capper Street, London, WC1E 6JB, UK

## Abstract

**Background:**

Improving the reproductive health of young women in developing countries requires access to safe and effective methods of fertility control, but most rely on traditional rather than modern contraceptives such as condoms or oral/injectable hormonal methods. We conducted a systematic review of qualitative research to examine the limits to modern contraceptive use identified by young women in developing countries. Focusing on qualitative research allows the assessment of complex processes often missed in quantitative analyses.

**Methods:**

Literature searches of 23 databases, including Medline, Embase and POPLINE^®^, were conducted. Literature from 1970–2006 concerning the 11–24 years age group was included. Studies were critically appraised and meta-ethnography was used to synthesise the data.

**Results:**

Of the 12 studies which met the inclusion criteria, seven met the quality criteria and are included in the synthesis (six from sub-Saharan Africa; one from South-East Asia). Sample sizes ranged from 16 to 149 young women (age range 13–19 years). Four of the studies were urban based, one was rural, one semi-rural, and one mixed (predominantly rural). Use of hormonal methods was limited by lack of knowledge, obstacles to access and concern over side effects, especially fear of infertility. Although often more accessible, and sometimes more attractive than hormonal methods, condom use was limited by association with disease and promiscuity, together with greater male control. As a result young women often relied on traditional methods or abortion. Although the review was limited to five countries and conditions are not homogenous for all young women in all developing countries, the overarching themes were common across different settings and contexts, supporting the potential transferability of interventions to improve reproductive health.

**Conclusion:**

Increasing modern contraceptive method use requires community-wide, multifaceted interventions and the combined provision of information, life skills, support and access to youth-friendly services. Interventions should aim to counter negative perceptions of modern contraceptive methods and the dual role of condoms for contraception and STI prevention should be exploited, despite the challenges involved.

## Background

Improving reproductive health is central to achieving the Millennium Development Goals on improving maternal health, reducing child mortality and eradicating extreme poverty [1,2]. This requires that women have access to safe and effective methods of fertility control. The promotion of family planning, so that women can avoid unwanted pregnancy, is central to the World Health Organisation (WHO) work on improving maternal health and is core to achieving the Millennium Development Goal on this [3].

In developing countries, maternal mortality is high, with 440 deaths per 100,000 live births (in sub-Saharan Africa, this figure reaches 920) [2]. One in three women give birth before age 20 and pregnancy-related morbidity and mortality rates are particularly high in this group [2]. One quarter of the estimated 20 million unsafe abortions and 70,000 abortion related deaths each year occur among women aged 15–19 years, and this age group is twice as likely to die in childbirth as women aged 20 or over [2]. It is estimated that 90% of abortion-related and 20% of pregnancy-related morbidity and mortality, along with 32% of maternal deaths, could be prevented by use of effective contraception [1]. In sub-Saharan Africa, it is estimated that 14 million unintended pregnancies occur every year, with almost half occurring among women aged 15–24 years [4].

Premarital exposure to pregnancy risk has increased, with a widening gap between sexual debut and age of marriage, and increased sexual activity prior to marriage [5,6], placing young women at increased risk when they are most socially and economically vulnerable. Reported sexual activity among adolescents in developing countries is generally high, although there is considerable variation between countries [5,7], and data validity is often poor [8]. In sub-Saharan Africa, 75% of young women report having had sex by age 20 [9].

However, few sexually active adolescents in developing countries use modern contraceptive methods such as oral contraceptives and condoms, and although there is considerable variation between countries, uptake is generally much lower than in developed countries [5]. For example, 69% of adolescent women in a UK study reported use of a modern contraceptive method at most recent sex, compared with 12% in Mali, and in the US 54% of 15–19 year old females reported condom use at most recent sex, compared with 21% in Tanzania [5]. Overall, it is estimated that 37% of unmarried, sexually active women aged 15–24 years in sub-Saharan Africa use contraception but only 8% use a non-barrier method [10]. Hubacher, Mavranezouli and McGinn [4] suggest that the choice of implant rather than oral or injectable contraceptives could have a big impact on unintended pregnancy in this age group. However, greater promotion of any modern method has to be informed by better understanding of why uptake is so low among adolescents in the first place.

Previously identified limits to contraceptive use among adolescents in developing countries include lack of knowledge, sex education and access to services; risk misperceptions; and negative social norms around premarital sexual activity and pregnancy [11,12]. Only one of these reviews focused on adolescents, and neither focused exclusively on qualitative research nor adopted systematic review methodology to critically appraise the included research studies. Focusing on qualitative research allows the assessment of complex processes, often missed in quantitative studies [13,14], and the assessment of study quality allows the selection of the most reliable and valid findings. This, in turn, improves the reliability and validity of the conclusions drawn.

Although there has been a systematic review of qualitative research on young people's sexual behaviour [15], we know of no systematic reviews of qualitative studies specific to contraceptive use to determine the full extent of the difficulties faced by young women in accessing modern methods. We undertook a systematic review of qualitative research on young women's own views of their contraceptive choices to examine factors limiting modern method use. By combining the findings from such studies we can demonstrate how themes may be common across settings and contexts.

## Methods

Table 1 shows the bibliographic databases, and journals, searched for literature from 1970–2006. We also searched the websites of a number of relevant reproductive health organisations. Citation searches of key authors were conducted. Searches combined adolescent, contraceptive, pregnancy, safe sex and choice terms (Table 1). Full details of the search strategy are available from the first author. We originally planned to include quantitative research in our review, but the high number of papers we sourced (19551 references from developing and developed countries, of which 4042 were potentially relevant) made this impossible. The review was refined to include only qualitative research (studies which used qualitative data collection methods, included the young women's views in their own words, and used qualitative methods of analysis).

**Table 1:** Review search strategy

**Databases**
ASSIA, ChildData, CINAHL, Cochrane Library (Central Register of Controlled Trials, Database of Systematic Reviews, Database of Abstracts of Reviews of Effects), COPAC (including British Library Catalogue), CSA websites, Digital Dissertations, EMBASE, ERIC, HDA, Health Technology Assessment Database, HEBS, HMIC, King's Fund Database, HELMIS, Index to Theses, MEDLINE, NHS EEDS database (via CRD), OCLC PapersFirst, POPLINE^®^, PychINFO, Sociological Abstracts, Web of Science – Social Science Citation Index/Science Citation Index, Websites (Omni.ac.uk, Sosig.ac.uk, Google.com, Association of Reproductive Health Professionals, Child Trends, fpa, International Women's Health Coalition, Planned Parenthood, Population Council, the Guttmacher Institute, United Nations Population Fund, and the World Health Organisation).

**Hand searches**

Journal of Family Planning and Reproductive Health Care (formerly British Journal of Family Planning), Contraception, Reproductive Health Matters, Journal of Adolescence, Journal of Adolescent Health, Perspectives on Sexual and Reproductive Health (formerly Family Planning Perspectives), and International Family Planning Perspectives.

**Search terms**

***Contraceptive terms***

Contraceptive/contraception, birth control, family planning/planned parenthood, pregnancy/pregnant, abortion, reproductive/reproduction, safe sex/unsafe sex/safer sex, protected intercourse/unprotected intercourse/unprotected sex/protected sex, sexual abstinence/abstain (include limit – sex), Depoprovera/Depo-Provera/Noristerat, Implanon/Norplant, intrauterine device (IUD)/intrauterine system (IUS)/coil, combined pill (oestrogen and progestogen)/progestogen only pill, diaphragm/cervical cap/spermicide – foam, jelly, cream, condom/Femidom/prophylactic/chemoprophylaxis/barrier method, morning-after-pill/Levonorgestrel/Yuzpe regimen, persona/fertility awareness/rhythm method, withdrawal/pulling out, douching

***Adolescent terms***

Adolescent/adolescence, teen/teenage/teenager, young woman/young women, young people/young adults, young people/young adults, young female, girl/girls
***Choice terms (used as limits where necessary)***
Choice/choose, uptake, use/nonuse/usage, (dis)continue/(dis)continued/(dis)continuing/(dis)continuation, acceptability/acceptance, utilise/utilize, attitude, knowledge, behaviour/behaviour, practice, switching, decision making, satisfaction

Table 2 shows the inclusion/exclusion criteria. Included studies were published between 1970 and 2006, focused on the 11–24 years age group and females (or presented female data separately from male), included qualitative research, and at least one quarter of the study's results were on uptake, use (or non-use), choice or discontinuation of contraceptives (including all non-permanent methods). Studies that focused exclusively on pregnancy, abstinence, sexual debut or sexual behaviour, and those published in languages other than English, were also excluded. The abstracts (or papers where no abstracts were available) of 4042 papers were reviewed and 3975 were excluded because they were not qualitative, had an irrelevant focus, topic or population, or did not include young women's own reports of use. In total, 47 studies were identified for inclusion in the review, 35 from developed countries and 12 from developing countries. The latter are the focus of this paper. Developing countries were defined as those in the areas of Africa, the Caribbean, Central America, South America, Asia excluding Japan and Hong Kong, and Oceania excluding Australia and New Zealand [16].

**Table 2:** Inclusion/exclusion criteria

**Criteria**	**Inclusion**	**Exclusion**
Date	1970–2006	Pre-1970
Age	11–24 years (11–19 years were the main focus, and studies were only included if data for the 11–19 year age group were shown separately).	Studies with focus on populations aged 25 years and over.
Sex	Female (or female data presented if studies include males and females).	Male only.
Study design	Qualitative research (studies which used qualitative data collection methods, include the young women's views in their own words, and have used qualitative methods of analysis). Interview, focus group, and participant observations studies.	Quantitative research (studies which used quantitative data collection methods, pre-determined or fixed responses, and quantitative methods of analysis). Editorials, literature reviews, book reviews, bibliographies, resource and policy documents, and methodological papers.
Contraceptive use	Uptake, use, non-use, choice or discontinuation of contraceptive use as main focus; user perspectives on individual contraceptive methods (including condom use for pregnancy prevention).	Studies focused exclusively on pregnancy, abstinence, age of sexual debut, number of sexual partners, HIV or other STI prevention, medical contraindications.
Contraceptive methods	All non-permanent contraceptive methods (contraceptive pill – combined and progestogen-only, injection, implant, IUD/IUS, male condom, female condom, diaphragm/cap, and spermicides). Studies on natural or traditional contraceptive methods (withdrawal, natural family planning, and periodic abstinence).	Studies of permanent solutions to fertility control (male and female sterilisation).
Measures and episodes of contraceptive use	Studies reporting contraceptive use at any episode (e.g. first or last sexual intercourse), of any timescale (e.g. last month, last three months, last year, ever), of consistent use (e.g. always, sometimes, never), and studies with dichotomous or multiple measures (e.g. any use or methods specified by respondents).	
Locations	All countries (studies from developing and developed countries were reviewed separately).	
Language	Written in English	All other languages
Relevance	One quarter to all of the study's results relate to contraceptive use.	Less than one quarter of the study's results relate to contraceptive use

There is considerable debate around the systematic review of qualitative research and the methods by which it should be appraised and synthesised [13,17-24]. As a result we used approaches that have been developed and published by researchers elsewhere. We employed quality assessment criteria based on those developed by Attree and Milton [25], Harden et al [26], and McDermott and Graham [27]. Studies were assessed on eight criteria relating to the extent to which they included: adequate description of their aims, background and context; clear detail on recruitment, the sample and data collection; and appropriate data analysis and interpretation (including presentation of sufficient original data and integration of data, interpretation and conclusions) (Table 3). Two of the authors (LW and AP) independently appraised each study, compared and discussed differences, and agreed on the final appraisal for each.

**Table 3:** Quality Assessment Criteria*

	**Criteria**
Aims	Clear statement of the aims or research questions of the study
Background	Explicit connection to existing theory or literature. Does the study include a comprehensive literature review? Is there sufficient explanation of, and justification for the study focus?
Context	Is the context/setting of the research adequately described? Are the circumstances under which the research was carried out reported?
Sampling/recruitment	Is there a clear description of the sample, including the size and characteristics of the sample, and how sampling and recruitment were conducted? Are exclusions and refusals accounted for or described?
Data collection	Is there a clear description of the research methodology used? This should include description of the means of data collection. Is how, as well as by whom, data collection was conducted reported?
Data analysis	Is there a clear description of the data analysis method and process? Again this should include description of how, and by whom, the analysis was conducted.
Data interpretation	Is there a clear discussion of the research findings? Does the study present sufficient original data to support the findings, and to demonstrate that these and the conclusions are grounded in the data? Is there clear integration of the data, interpretation and conclusions? Are the study context and sample considered in the findings?
Reliability/validity	Is there evidence that the reliability and validity of the analysis has been addressed?

*Adapted from McDermott and Graham (2005) [27].

Meta-ethnography is one method of qualitative data synthesis [28], and a number of reviews using this approach have been published [25-27,29-32] so this was the approach we adopted. Meta-ethnography allows the development of a broader perspective while maintaining the specificity of individual studies. In interpreting rather than simply aggregating the data, a re-conceptualisation of key themes aims to synthesise and extend the findings of individual component studies [28].

This process of synthesis requires repeat reading and constant comparison of each of the studies included in the review. Studies were first reviewed to identify key themes, keeping the study context in mind. These were then organised in tabular form to compare themes across studies. By identifying the key themes, the studies can be translated into one another by examining their similarities and contradictions. Then, to answer the review question about limits to modern contraceptive use reported by young women in developing countries, a 'lines-of-argument synthesis' was developed. In this, the similarities and differences in the findings of individual studies were examined to develop a new, broader understanding of the issue, which goes beyond, but maintains the specificity of, each individual empirical study [28].

## Results

Of the 12 studies from developing countries which met the inclusion criteria, five did not include sufficient original data to support their authors' interpretations, discussions and conclusions and were excluded [33-37].

The characteristics of the seven studies included in the synthesis are shown in Table 4[38-44]. Of these, six were from sub-Saharan Africa (two from South Africa, two from Tanzania, and one each from Nigeria and Mali) and one was from South-East Asia (Vietnam); five included only 13–19 year olds; and three included only females. Sample sizes ranged from 16 to 149 with 387 young women in total (there were also five further focus group discussions (FGD) for which the number of participants was not reported). Four of the studies were urban based, one was rural, one was semi-rural, and one was mixed (predominantly rural). The majority of the young women (where reported) were sexually active and unmarried. Two studies focused solely on a particular high-risk group of young women who had experienced abortions [39,41].

**Table 4:** Characteristics of the studies included in the data synthesis

**Study Author (date)**	**Aim**	**Sample size**	**Sample characteristics**	**Context/setting**	**Data collection**
Castle (2003)	To investigate the belief that hormonal contraceptives lead to long-term sterility	75 (+ 8 health professionals)	39 female, 36 male Most aged 15–19 years* Most educated (14 of 52 clients/non-clients had no formal education) Most sexually active* Most unmarried*	Bamako and Sikasso, Mali (urban) Mix of clients of peer education programme, peer educators, and non-clients from same areas	Individual interviews
Kiluvia & Tembele (1991)	To learn about Tanzanians' opinions, knowledge and behaviour with respect to family planning and child spacing	141 in 16 focus group discussions (FGD)	63 male and 78 female Aged 15–19 yrs All participants had either some primary schooling or no schooling at all Most not sexually active* Most unmarried*	9 villages in 6 districts of Tanzania (Kisarawe, Mwanga, Dar es Salaam (urban), Sumbawanga, Dodoma, Songea) (all but one rural)	Focus group discussions
Nguyen, Liamputtong & Murphy (2006)	To examine young people's knowledge and practice of contraceptives	16	12 female, 4 male Aged 15–24 years Education – secondary (4), high school (5) and college/university (7) Females – all unmarried & all sexually active	Ho Chi Minh City, Vietnam (urban) Hospital based sample (had experienced abortion)	Individual interviews
Otoide, Oronsaye & Okonofua (2001)	To examine attitudes and beliefs of abortion and contraception	149 in 20 FGD	All female Aged 15–24 years Education – tertiary (23), secondary (79), primary/secondary (19), primary (7), none/primary (15), none (6) 116 sexually active Marital status not reported	Benin City, Nigeria (urban) Sample were selected from a range of areas of residence	Focus group discussions
Rasch et al (2000)	To understand the experiences of adolescent girls with illegally induced abortion	51	All female Aged 15–19 years 25 still in school (rest employed as house-girls, waitresses or engaged in petty trade) All sexually active All unmarried	Dar es Salaam, Tanzania (urban) Hospital sample (pregnant and admitted to hospital with incomplete, induced abortion)	Individual interviews
Richter & Mlambo (2005)	To explore and describe perceptions of teenage pregnancy	32	22 female, 10 male Aged 13–19 years Education not reported Marital status not reported At least 10 females sexually active* (sampled from ante/post-natal clinics)	Bushbuckridge district of Limpopo Province, South Africa (rural) Sample from an antenatal clinic, a family planning clinic, a postnatal ward, and a Love-Life Youth Centre	Individual interviews
Wood & Jewkes (2006)	To collect information to improve access to and quality of contraceptive services for adolescent girls	35 (interviews) & 5 FGD (+ 14 nurses – interviews & focus groups)	All female Aged 14–20 years Education not reported 33 interviewees were sexually active† All interviewees were unmarried†	Limpopo Province, South Africa (semi-rural) Sample from semi-rural areas surrounding the main town – recruited from clinic waiting rooms or schools*	Individual interviews and focus group discussions

* Actual figures not reported.

† Not reported for focus groups.

Table 5 shows the themes identified in each paper (ticks indicate that the theme was addressed in the paper and crosses indicate that it was not). In the 'lines-of-argument synthesis' these were translated into five main categories, presented here and illustrated with representative quotes from the original papers.

**Table 5:** Limits to use of modern contraceptive methods: themes identified in the papers in the synthesis

	Castle (2003)	Kiluvia & Tembele (1991)	Nguyen, Liamputtong & Murphy (2006)	Otoide, Oronsaye & Okonofua (2001)	Rasch et al (2000)	Richter & Mlambo (2005)	Wood & Jewkes (2006)
Misperception of pregnancy risk	✓	X	✓	✓	✓	✓	✓
Knowledge of modern contraceptive methods	✓	✓	✓	✓	✓	✓	✓
Reliance on traditional contraceptive methods or abortion	X	✓	✓	✓	✓	X	✓
Access to modern contraceptive methods	✓	✓	X	✓	✓	✓	✓
Concerns over side effects of modern contraceptive methods	✓	✓	✓	✓	✓	✓	✓
Desire of pregnancy/fertility proof	✓	X	X	✓	✓	✓	✓
Partner influence/control	✓	✓	✓	✓	✓	✓	✓
Unplanned/forced sex	X	X	✓	X	✓	✓	X
Social/economic influences and pressures	✓	✓	✓	X	✓	✓	✓
Threat to reputation	✓	✓	✓	✓	✓	✓	✓

✓ – Theme addressed in the paper.

X – Theme was not addressed in the paper.

### 1. Knowledge of pregnancy risk, prevention, and access to modern contraceptives

The majority of young women in each study reported receiving little sex or contraceptive education from parents, health services or elsewhere. Any education they did receive often simply reinforced common misperceptions of modern contraceptives [38,39,43,44]. The synthesis highlights that the young women had inaccurate perceptions of pregnancy risk, including changes over the monthly cycle. Many thought that they could not get pregnant at first sex, or if they had sex standing up or infrequently [38,39,41-44].

Even though young women were generally aware of modern hormonal contraceptive methods, all the studies suggested that young women had limited knowledge of how they worked or how to use them properly. There was a general misperception that you only had to take the pill when having sex:

"I take a pill when I know my boyfriends is coming and we probably going to make love. I sometimes forgot to take it before we make love so I take it after we made love." (Young woman, South Africa, page 65) [43].

In Mali, young women suggested that access to modern methods was not a problem [38], but health services were considered inaccessible in most of the studies. This was partly because of the distance to these [40,43], but mainly because young women perceived services to be catered principally for married women and they had significant fears of receiving a negative reception from clinic staff [39,40,42-44]:

"We just feel ashamed but we don't challenge them. Sometimes you may challenge them and find that you use words which may hurt them, then the next time you go there, you find they refuse to help you." (Young woman, South Africa, page 113) [44].

In contrast, some described being pressurised by mothers and nurses to start contraception or use particular methods [44]. This was rarely accompanied by proper guidance on use, which the young women often felt unable to request:

"There is nothing explained to us, it's just go through, what method do you want and if it's an injection they will inject you. The nurses always look busy and we are afraid to ask questions." (Young woman, South Africa, page 67) [43].

Although difficulties accessing contraceptives was not directly addressed in the Vietnamese study, it was clear that young women were rarely provided with adequate information about modern contraceptives, or even sex in general, which was considered too sensitive a topic to talk about, and only two of the twelve young women in the study had ever used a modern method [41].

Given the reported barriers to service use, access to contraceptives was therefore often through unofficial or commercial channels:

"How person go dey go UBTH because of family planning, if you enter any chemist you will get family planning. [Why would someone go to a tertiary care centre in the city because of contraception? If you enter any patent medicine store you will get contraceptives.]" (16 year old out of school female, Nigeria, page 79) [42].

Although these outlets were thought more discreet and confidential, contraceptive provision was unlikely to be accompanied by accurate information on use or side effects [42]. The expense could also be prohibitive, and one study described how Anna (Tanzania), "...needed all her money for her daily needs" (Page 58) [39].

Condoms, which were available from numerous sources, were also considered to be an appropriate and accessible modern method by some young women:

"If the partner of the girl likes using the condom, it is more appropriate because with the condom there is no possibility of a mistake, access is easy, and the price is affordable." (18 year old woman with secondary schooling, Mali, page 190) [38].

However, they were not the contraceptive method most would opt for because they were more often thought to be for HIV/STI prevention, not pregnancy prevention:

"People said that using condoms was to prevent getting pregnant... But I did not know very well about this issue. I just knew that condoms were used mainly for preventing from getting AIDS." (Cuc, 18 year old female high school student, Vietnam, page 406) [41].

### 2. Hormonal contraceptive side effects, menstrual disruption and fertility fears

All the studies showed that concerns over experienced and perceived side effects of hormonal contraceptive methods, particularly menstrual disruption, were central to young women's non-use of these. Fear of infertility was the most often cited:

"...women who use contraceptives will find it difficult to conceive when they eventually get married." (20 year old female youth club member, Nigeria, page 80) [42].

In Mali and South Africa, menstruation represented the womb being cleared of "dirt" [38,44], and was equated with good health [38,41,44]. Therefore, methods which interrupted the perceived natural pattern of menstruation were unacceptable:

"If you're not on the injection the blood stays somewhere next to the womb, and if you don't conceive that month, the blood can get out; but if you use Nur-Isterate [injection], this blood doesn't pass easily to the place next to the womb, and it means your body will never be as it was before, and this blood prevents you from ever falling pregnant." (Young woman, South Africa, page 113) [44].

### 3. Partner relationships, sex and pressure

All of the studies made reference to partners' attitudes towards contraception, which were often crucial. Partners were reported to manipulate, force, threaten, and use violence to get young women not to use contraception [39,41,43,44]. This was particularly the case with condoms, which some of the young women's partners did not want to use because they reduced sexual pleasure [39,41,43]:

"I asked my boyfriend to use condoms but he did not accept it, and he used withdrawal... I knew many contraceptive methods. But using them was up to my boyfriend. He did not like using them. I had to follow him." (Ha, 19 year old college student, Vietnam, page 411) [41].

However, partner control over other contraceptive methods was also apparent [40,44]. Some of the girls interviewed by Wood and Jewkes [44] reported being encouraged by their male partners to use contraception, but others reported that their partners wanted them to get pregnant to prove the partners' fertility. Wood and Jewkes state:

"Some boyfriends reportedly demanded that contraception be stopped, at times threatening to use, or using, physical violence to enforce this, or tearing up the clinic card and throwing away the pills." (page 111) [44].

In South Africa, partners' control over contraceptive use appeared strong when they provided gifts or money in exchange for sex [43]. However, two African studies [39,43] suggested that some young women wanted to become pregnant as a bargaining tool to solidify relationships:

"I fell pregnant because I wanted my boyfriend to marry me." (Young woman, South Africa, page 66) [43].

### 4. Reputations and social status

All the studies identified young women's reputations and social status as a limit to contraceptive use. The synthesis indicates that there was considerable social disapproval of premarital sex and pregnancy:

"Community members do not accept teenage pregnancy they even scold us randomly when they meet a pregnant girl." (Young woman, South Africa, page 64) [43].

For many, accessing contraceptives, particularly by going to a clinic, constituted a public admission of having had sex, and was linked to being promiscuous or a prostitute [39-41,43,44].

The side effects of hormonal contraceptive methods also constituted a significant threat to personal reputation:

"It [amenorrhea] is abnormal. When a woman cannot have children she is called a lot of names like "whore" and people say that she must have had a lot of abortions." (19 year old young women at university, Mali, page 195) [38].

In both West and Southern Africa, future social status was highly dependent on future fertility, concerns about which led to a fear of being "condemned" (Page 111) [44]. This was reinforced by the young women's partners, their parents and wider society:

" [If you are thought to be infertile] you won't be loved, especially if you have a mother-in-law who wants grandchildren. If you have a co-wife, at every opportunity she will boast that she has children and you don't. There are men who follow their parents blindly. If in your marriage you have a mother-in-law who asks her son to divorce you, he will do it without even thinking." (19 year old female with Franco-Arabic schooling, Mali, page 195) [38].

Despite the expressed disapproval of premarital sexual activity, getting pregnant offered definitive proof of childbearing potential. In South Africa, this was one of the few available means of attaining respected status [44], particularly among less educated women who often lacked alternatives [43,44].

### 5. Reliance on traditional contraceptive methods and abortion

As a result of the difficulties with modern contraceptive methods discussed above young women were more likely to rely on traditional methods, such as periodic abstinence, withdrawal, or charms and herbal mixtures from traditional healers [40-42,44]. However, the studies in this review specifically focused on modern methods, so use of traditional methods was not discussed in detail. In one study, even common medicines such as aspirin or antibiotics were thought effective contraceptives:

"How woman fit prevent belle? Na many ways. She fit use quinine... [How can a women avoid pregnancy? There are many ways. She can take quinine (anti-malarial drug)...]." (17 year old uneducated female hairdresser, Nigeria, page 79) [42].

Relying on traditional methods provided greater privacy:

"In using modern methods, one has to go to UMATI [Family Planning Association of Tanzania] and many people will know, while in using local herbs it's one's own secret." (Female in 15–19 years age group, Tanzania, page 10) [40].

Alternatively, in Tanzania and Nigeria abortion was recognised as a fertility control option if an unintended pregnancy occurred [39,42], even though illegal (in these countries and Mali abortion is only permitted to save a woman's life). In one study, the potential side effects of abortion, and the threat to fertility it incurred, were not thought as serious, or as likely, as those of modern contraceptive methods:

"One D and C [abortion] is safer than 16 packs of daily pills...many girls say this." (22 year old female undergraduate, Nigeria, page 80) [42].

## Discussion

This systematic review of qualitative research demonstrates that young women's use of modern contraceptive methods in five developing countries is limited by a range of factors, which centre on lack of knowledge, obstacles to access, and lack of control. Use of hormonal methods was limited because of lack of knowledge and access and concern over side effects, especially fear of infertility. Although often more accessible, and sometimes more attractive than hormonal methods, use of condoms was limited by their association with disease and promiscuity and greater male control of this method. As a result young women often relied on traditional methods or abortion. This is similar to findings elsewhere [11,12,45]. This paper adds to the literature by demonstrating that the limits could be common across very different settings and contexts. Our findings are also strengthened by the inclusion of only methodologically rigorous studies: five papers had to be excluded for not reporting enough original data to support their findings.

However, it is important to note that the review is limited to five countries; four in sub-Saharan Africa (Mail, Nigeria, Tanzania and South Africa) and one in South-East Asia (Vietnam). Although the overarching themes were common across all locations and settings, we do not mean to suggest that conditions will be homogenous for all young women in all developing countries. The findings of the South-East Asian study were largely consistent with those from sub-Saharan Africa, and similarities are apparent in other South American and South Asian studies [11,46-49], although these have not focused exclusively on adolescents or qualitative research. This is similar to findings reported elsewhere in a larger review of young people's sexual behaviour [15]. There were also commonalities across urban and rural locations and across education levels (where reported), although differences between these have previously been reported [9,45]. However, the limited geographical range of the studies in this review and the lack of comparable research in this field from others areas (particularly from Central and South America, Asia and Oceania) should be noted. Further research is necessary to examine the restrictions on contraceptive use in these areas.

It is also important to note that our review is limited to a small number of papers and to ask whether we succeeded in identifying all relevant qualitative research. Qualitative research terms have been successfully incorporated into review search strategies [27], but we did not use these because we originally planned to include quantitative research. It is possible that a more specific search strategy could have identified further qualitative papers of direct relevance to the review question, but we are doubtful that this is the case. Our search strategy used an extensive range of search terms (see Table 1) and identified almost 20000 references. After an initial review of these for relevance on contraceptive use, 4000 references were specifically screened: firstly for direct relevance to the review question, and secondly, for qualitative research. However, we concede that the exclusion of non-English language studies could have missed some relevant papers, and that there could be further qualitative studies published in books or the grey literature, which have been missed (although our strategy did include searches of the Copac Academic & National Library and the British Library Catalogues, as well as attempts to source grey literature from the websites of relevant organisations such as the WHO and the Population Council). Also, our inclusion criteria required that at least one quarter of the study's results were on uptake, use (or non-use), choice or discontinuation of contraceptives. It is possible that further studies, particularly in the areas of teenage pregnancy and childbearing, could have touched upon such issues but been excluded from the review because of this criterion (or because the study abstract, on which most exclusions were based, did not refer specifically to contraception). However, efforts to increase the uptake of modern contraceptives require detailed understanding of the barriers to this; as is provided by the studies included in this review.

Contraceptive choice among the young women in the African and Vietnamese studies in this review was restricted by their concerns about fertility and their status as women. Studies show that social disapproval of premarital sex, particularly when young women are still at school, limits young women's knowledge of, and access to, health services [12,45,50]. This was also the case in the studies we reviewed. Misconceptions of modern hormonal methods, particularly that their use could cause infertility, were common among the young women in this review. Such beliefs are often reported to be community-wide [11,12]. Young women are under pressure to remain fertile so that they can bear children, once it is socially acceptable to do so. Bearing children is necessary to be respected within their husband's family, protect from divorce, and provide economic support or insurance for the future; conversely, not being able to bear children poses a significant threat to social and economic survival [11,12,45,48,49,51]. So for young women, preserving future fertility becomes as important as preventing pregnancy, and condoms and traditional methods, which do not threaten fertility, are relied on. Even abortion was sometimes viewed as more appropriate for a few of the young women in this review. Although illegal and dangerous in most sub-Saharan African countries, it could be seen as more accessible (and less risky) than accessing or using hormonal contraceptives [52,53].

Promotion of modern contraceptives requires multifaceted interventions at all levels of society. Use of modern contraceptive methods has been successfully promoted for child spacing and limiting family size among older married women with children in developing countries [1,54], but there is still considerable variation between countries. In two of the countries in this review (South Africa and Vietnam), uptake is high (55% and 57% of currently married 15–49 year old women report using a modern method respectively) [55]. However, in the remaining three African countries, uptake is low (7% in Mali, 8% in Nigeria and 17% in Tanzania) [55], despite evidence of unmet need (29% in Mali, 17% in Nigeria and 22% in Tanzania) [55]. It is questionable that attempts to increase use of modern methods among adolescents will be effective in countries where they remain unacceptable to older married women. Increasing uptake among all women will require countering the negative perceptions of modern contraceptive methods, and challenging inaccurate beliefs and cultural norms around fertility at the community-level. In addition, increasing uptake among young, unmarried women, for whom the health risks and consequences of unplanned pregnancy are of particular concern [2], will require the provision of more targeted promotion of life skills, support and access to youth-friendly services for adolescents [5,56].

Although this paper focused on women, the considerable, sometimes coercive, role that men have in contraceptive decisions is apparent [12,45], particularly when sex is accompanied by financial or material reward [50,52]. The findings from young men, where included in the studies reviewed here, confirmed those obtained from the perspectives of young women [38,40,41,43]. This highlights the need to include both sexes in sexual and reproductive health interventions [57], particularly in relation to condom use.

The role of HIV/STIs in young women's sexual attitudes and experiences was beyond the scope of this review, but condoms were often considered accessible, and in some cases more attractive than other modern contraceptive methods, even though their association with disease, promiscuity, and commercial sex can limit use [12,15,45,57]. Cultural acceptability of condoms for HIV prevention has increased [58-60], as has use among young, unmarried women [59,61]. Fear of unwanted pregnancy may be as much a motivation for condom use as fear of AIDS [59], and the dual pregnancy and HIV prevention role of this method should be capitalised on. The promotion of condoms for HIV and STI prevention is essential, especially in areas of high HIV prevalence, but their promotion for pregnancy prevention should also be actively encouraged. Given women's concern with future fertility, it should be stressed that it can be enhanced by protecting against STIs. Clearly the dual use of condoms would be facilitated by greater integration of HIV and reproductive health services [62].

## Conclusion

Although limited to five countries, this systematic review of qualitative research suggests that similar factors restrict young women's contraceptive choices across different developing countries, in particular limited knowledge, access, worries about fertility and the low status of women. With high rates of pregnancy-related morbidity and mortality among adolescent women [2], and the clear role of improving reproductive health in achieving the Millennium Development Goals on maternal health, child mortality and poverty [1,2], the consequences of not improving access to modern contraceptive methods are dire.

Global variations in sexual behaviour exist and no one sexual or reproductive health intervention will work everywhere [63]. However, our finding that major limits to modern contraceptive use could be common across various settings, contexts and countries supports the potential transferability of successful interventions, given appropriate adaptation to local socio-cultural conditions [64]. To date, adolescent reproductive health interventions in developing countries have had modest positive effects on contraceptive behaviour. Increasing modern contraceptive method use requires community-wide, multifaceted interventions, which should aim to counter negative perceptions of modern methods. Opportunities for intervention are apparent, particularly in relation to the dual use of condoms for pregnancy and HIV prevention, and should be capitalised on.

## Competing interests

The authors declare that they have no competing interests.

## Authors' contributions

LW, GH and MP designed and initiated the study. LW and AP critically appraised and reviewed papers for inclusion in the review, and conducted the data synthesis. LW prepared the first draft of the paper. All authors made substantial comments and contributions to subsequent drafts and approved the final version submitted for publication.
